# Cross-protection conferred by intradermal PRRSV-1 modified live vaccine against a highly virulent Vietnamese PRRSV-2 strain in pigs

**DOI:** 10.3389/fvets.2026.1866378

**Published:** 2026-07-08

**Authors:** Bui Thi To Nga, Salvador Romero-Aguilar, Ngo Thi Ngoc Tram, Nguyen Thi Lua, Tran Ngoc My Linh, Vo Hong Thao Nguyen, Nguyen Thi Hoa, Massimiliano Baratelli, Do Tien Duy

**Affiliations:** 1 Faculty of Veterinary Medicine, Vietnam National University of Agriculture, Hanoi, Vietnam; 2 HIPRA S.A., Girona, Spain; 3 Faculty of Animal Science and Veterinary Medicine, Nong Lam University HCMC, Ho Chi Minh, Vietnam; 4 The Animal Biomedical Research Laboratories, Nong Lam University HCMC, Ho Chi Minh, Vietnam

**Keywords:** intradermally, pig, porcine reproductive and respiratory syndrome virus type 2, vaccine, Vietnam

## Abstract

Porcine reproductive and respiratory syndrome (PRRS) remain one of the most economically significant diseases affecting the global swine industry. Caused by the PRRS virus (PRRSV), the disease leads to reproductive failure in sows and respiratory disorders in pigs of all ages. In Vietnam, PRRSV was officially reported in 2007, and the emergence of highly pathogenic PRRSV (HP-PRRSV) strains has since intensified outbreaks with high morbidity and mortality. This study evaluated the efficacy of the intradermal PRRSV-1 commercial vaccine (UNISTRAIN® PRRS, HIPRA) in piglets experimentally challenged with a Vietnamese HP-PRRSV-2 strain. Twenty-eight PRRS naïve, 21-day-old piglets were randomly assigned to a VC group, including vaccinated and challenged pigs (*n* = 12); UVC group, including unvaccinated and challenged pigs (*n* = 12); and a UVUC group, including unvaccinated and unchallenged pigs (*n* = 4). At 5 weeks post-vaccination, all animals in VC group and UVC group were challenged intramuscularly with 3 mL of HP-PRRSV (lineage L8E, 2025; at a dose of 10^5^ CCID₅₀/pig) and monitored for 28 days post-infection (dpi). Clinical signs, rectal temperature, viremia, antibody response, macro/micropathological lesions and growth performance were evaluated. Vaccinated pigs exhibited a lower incidence of fever and milder respiratory signs than unvaccinated pigs, corresponding to reduced clinical scores. Despite viremia in both groups post-infection, vaccinated pigs exhibited reduced viral loads, shorter viremia duration, and lower incidence than unvaccinated pigs at 7 dpi (*p* < 0.05). This constituted a significant reduction in viremia, evidenced by a 38.58% decrease in AUC. The antibody response developed earlier and persisted longer in vaccinated pigs, which displayed significantly higher S/P ratios at 7 dpi (*p* < 0.001). Lung lesions in vaccinated pigs were characterized by mild interstitial inflammation with preserved alveolar structure, whereas unvaccinated pigs exhibited severe, diffuse interstitial pneumonia with hemorrhage. Average daily gain was numerically higher in vaccinated animals, though not statistically significant (*p* > 0.05). Overall, intradermal PRRSV-1 vaccination partially protected against heterologous HP-PRRSV-2, lowering disease severity and promoting early antibody production.

## 1 Introduction

Porcine reproductive and respiratory syndrome (PRRS) is a major infectious disease of swine, characterized by reproductive failure in sows and respiratory disorders in growing pigs (1–3). Since its emergence, PRRS has become one of the most economically significant diseases affecting the global swine industry (4–7). Based on substantial genetic and antigenic divergence, the causative agent PRRS virus (PRRSV), is classified into two distinct species: Betaarterivirus suid-1 (formerly European type, PRRSV-1) and Betaarterivirus suid-2 (formerly North American type, PRRSV-2), which share only 50–70% nucleotide identity (8–12). Generally, PRRSV-2 isolates are considered more pneumo-virulent than PRRSV-1 (13), although several highly virulent PRRSV-1 strains have also been described (14–17).

In Vietnam, PRRSV-2 strains of North American origin are predominantly responsible for field outbreaks (18, 19). All isolates collected between 2007 and 2015 were assigned to sub-lineages 8.7 and 5.1 (18). More recent surveillance studies revealed that highly pathogenic PRRSV (HP-PRRSV) strains continue to dominate, particularly those belonging to lineage 8, sub-lineage 8E, followed by NADC-like lineage 1.4 viruses that were first detected in Vietnam, and a minority of isolates genetically related to vaccine strains (19, 20).

Vaccination remains the most practical and cost-effective approach for PRRSV prevention and control (21). However, the extensive genetic variability among PRRSV strains (22–25) frequently limits vaccine efficacy, which is typically high against homologous strains but inconsistent against heterologous strains (26–30). The degree of cross-protection has been shown to inversely correlate with the genetic distance between the vaccine and challenge strains (31), emphasizing the importance of evaluating each vaccine’s performance under relevant field and experimental conditions. Vaccination with a PRRSV-1 modified live vaccine (MLV) has been shown to provide partial protection against heterologous challenge with HP-PRRSV-2 (sub-lineage 8.7), alone or in combination with PRRSV-1 clade A, subtype 1 (32). Current modified live PRRSV vaccines can reduce clinical disease, viremia, and lung lesions; however, complete cross-protection against heterologous strains is rarely achieved because of the extensive genetic and antigenic diversity of PRRSV. We hypothesized that intradermal administration of a commercial PRRSV-1 modified live vaccine would induce protective immune responses and confer cross-protection against a heterologous highly pathogenic PRRSV-2 strain. Therefore, the objective of this study was to evaluate the efficacy of a commercial PRRSV-1 MLV vaccine administered intradermally against challenge with a contemporary HP-PRRSV-2 isolate (lineage L8E, 2025) by assessing clinical performance, virological parameters, immune responses, and pathological lesions.

## 2 Materials and methods

### 2.1 Ethical statement

This study was conducted in accordance with institutional guidelines for the care and use of laboratory animals and was approved by the Ministry of Agriculture and Rural Development (MARD), Vietnam (TCVN 8402:2010). All animal procedures performed in this study were reviewed and approved by the Animal Ethics Committee for the Use of Animals in Research of Nong Lam University (AEC-NLU), under approval number NLU251117.

### 2.2 Experimental design

Twenty-eight 21-day-old clinically healthy piglets were obtained from a high-health-status farm. All animals tested negative for ASFV, PRRSV, CSFV, and PCV2 by real-time PCR and ELISA before the study. After transportation to a biosafety level 2 (BSL2) facility, piglets were block-randomized by initial body weight; the personnel recording clinical/pathology endpoints were blinded to group allocation via coded ear tags and pen IDs into three groups: VC group, including vaccinated and challenged pigs (*n* = 12); UVC group, including unvaccinated and challenged pigs (*n* = 12); and a UVUC group, including unvaccinated and unchallenged pigs (*n* = 4). VC group was actively immunized with UNISTRAIN® PRRS ID vaccine (Hipra, Spain) which contains the PRRS-1 modified live strain VP-046. A single dose of 0.2 mL of the vaccine was administered by intradermal route at 21-day-old using the needle free administration device Hipradermic®, according to the manufacturer’s instructions.

**Figure 1:**
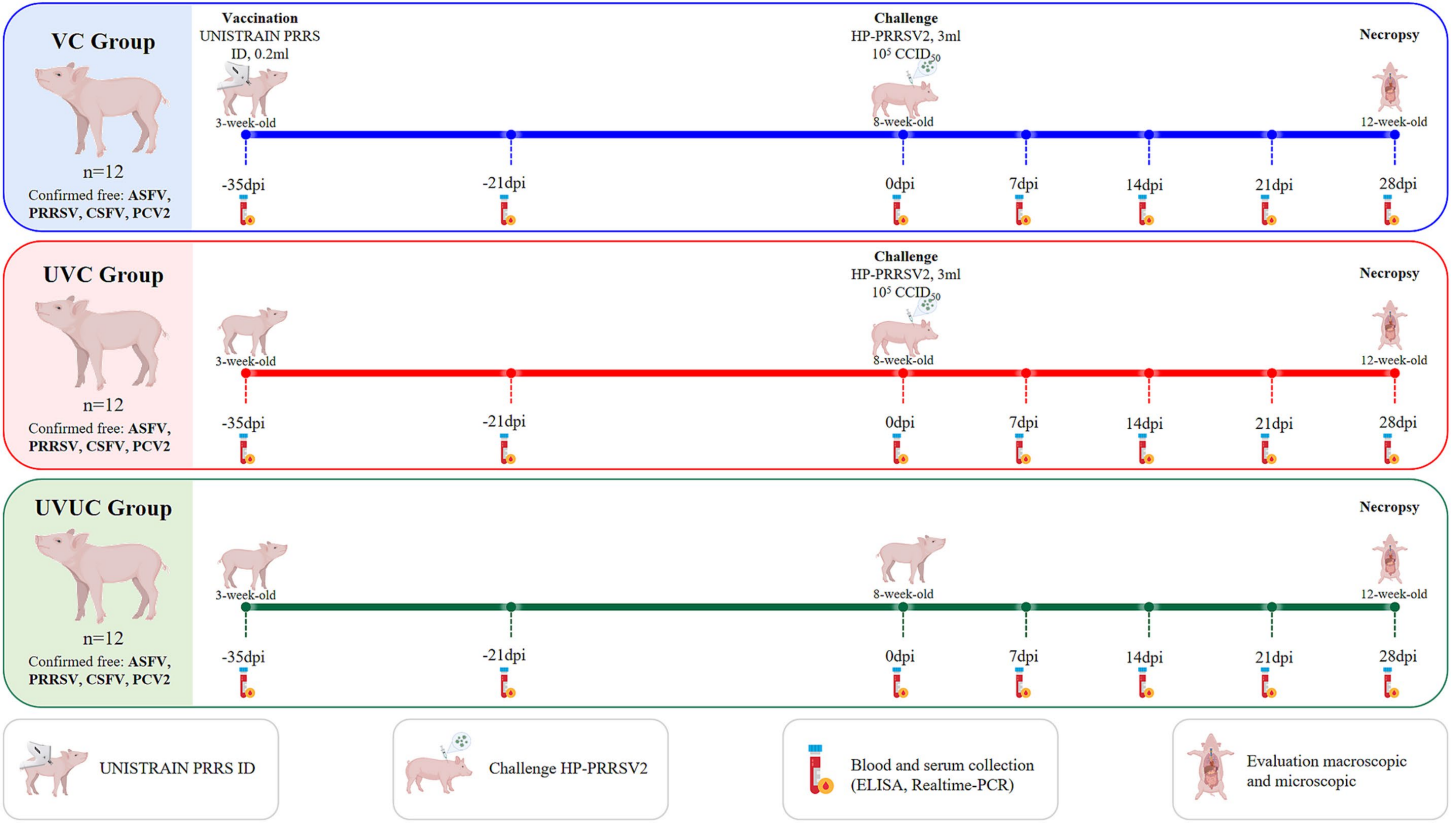
Schematic representation of the experimental design. ASFV, African swine fever virus; dpi, days post-infection; PCV2, Porcine circovirus type 2; PRRSV, Porcine respiratory and reproductive syndrome virus; CSFV, Classical swine fever virus; CCID50, 50% cell culture infectious dose; ID, Intradermal; VC, Pigs were vaccinated and challenged; UVC, Pigs were un-vaccinated and challenged; UVUC, Pigs were un-vaccinated and un-challenged.

Five weeks after vaccination (35 days post vaccination), all pigs in VC and UVC groups were challenged intramuscularly with 3 mL of HP-PRRSV (lineage L8E, 2025, accession number PV833346) at a dose of 10^5^ CCID₅₀/pig (33–35). The challenge strain shares 38.69% genetic homology with the PRRSV-2 vaccine strain used in this study. Pigs were housed in separate rooms under identical conditions, with the same feeding regime and water ad libitum. Clinical signs, viremia, antibody responses, and body weight were monitored at −35, −21, 0, 7, 14, 21 and 28 days post-infection (dpi) to evaluate the vaccine efficacy and growth performance. Pathological examinations were conducted at the end of the study. The experimental design is described in Figure 1.

### 2.3 Virus

**Figure 2:**
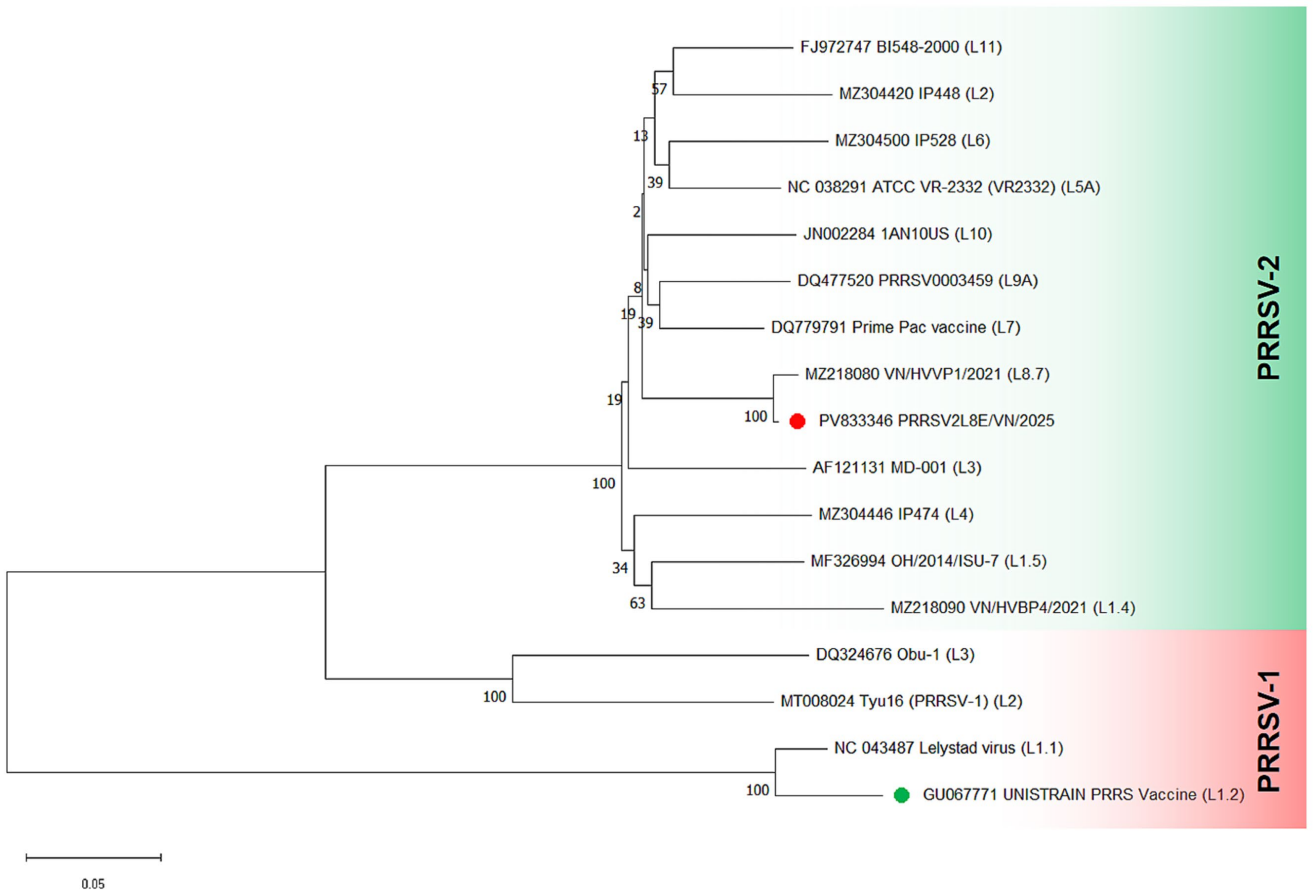
Phylogenetic relationships of the Vietnamese HP-PRRSV-2 isolate (●) and vaccine strains used in this study (●) based on ORF5 gene sequences. Phylogenetic analysis was performed in MEGA11 using the Neighbor-Joining method with *p*-distance model and 1,000 bootstrap replicates.

In this study, a HP-PRRSV type 2 isolate, accession number PV833346, collected in Vietnam in 2025, was employed as the challenge strain. Phylogenetic analysis based on the ORF5 gene classified the isolate into lineage 8, sub-lineage E (L8E). The isolate was characterized as highly pathogenic PRRSV2 based on its phylogenetic clustering with previously reported HP-PRRSV strains and the presence of characteristic molecular signatures, including discontinuous deletions in the NSP2 region (36), which are commonly associated with enhanced virulence. Meanwhile, the vaccine strain is classified as genotype 1, phylogenetically distinct from the challenge virus. Phylogenetic trees were constructed using the Neighbor-Joining (NJ) method with the p-distance model, and bootstrap analysis was performed with 1,000 replicates to evaluate tree robustness (Figure 2).

### 2.4 Clinical evaluation

The clinical state of the animals and the end-point criteria were evaluated by scoring the PRRSV-compatible clinical signs following a previously reported guide (11, 37). The clinical signs included pyrexia, body condition, respiratory, skin manifestations, neurological, mortality and other possible clinical signs. Specifically, clinical scoring was performed daily by trained veterinarians experienced in swine clinical assessment, using coded animal identification to ensure blinding to group allocation. The same evaluators conducted all clinical observations throughout the study to minimize inter-observer variability and reduce potential observation bias.

### 2.5 Pathological examination

All pigs were euthanized at 28 days post-challenge with the HP-PRRSV-2 strain and subjected to complete necropsy examination. Gross lesion scoring and histopathological evaluation of the lungs, lymph nodes, and other visceral organs were performed according to previously described methods. For blinded pathological assessment and consistent presentation of lesion findings, pigs in the vaccinated-challenged (VC) group were designated as A01–A12, whereas pigs in the unvaccinated-challenged (UVC) group were designated as B01–B12.

### 2.6 PRRSV real time-RT PCR

PRRS viremia was measured in serum of studied piglets by Realtime RT-PCR as follow. A total of 196 samples from the 28 piglets in each group had their serum samples collected at −35, −21, 0, 7, 14, 21 and 28 dpi. RNA was extracted from the serum samples using the GeneJET™ Viral DNA and RNA Purification Kit (Thermo Scientific, Lithuania) according to the manufacturer’s instructions. Viral load in blood was quantified by using a commercially available Realtime-PCR kit (Kylt® PRRSV NA/EU, AniCon Labor GmbH, Germany) according to the manufacturer’s instructions. The interpretation of results was based on the cycle threshold (Ct) values obtained during amplification. Samples with a Ct value ≤ 42 were considered positive for PRRSV RNA.

### 2.7 PRRSV serology

Anti PRRSV antibodies were determined from serum according to a commercial ELISA kits (IDEXX PRRS Ab Test) of experimental pigs collected at −35, −21, 0, 7, 14, 21 and 28 dpi. S/P ratio > = 0.4 was considered positive.

### 2.8 Growth performance

Weaned piglets were weighed at the start of the experiment (−35 dpi) and at the end of the experiment (28 dpi). Body weigh was calculated early in the morning before feeding. The average body weight (BW) was calculated using the formula: BW = Total body weight of piglets at the end of the trial/Number of piglets in the pen at the end of the trial. The average daily gain (ADG, g/pig/day) was calculated using the formula: ADG = Total weight gain of piglets during the experimental period/Total number of rearing days *1000.

### 2.9 Statistical analysis

Raw data were initially processed using Microsoft Excel 2016 and subsequently analyzed with GraphPad Prism 8. Viremia was quantified as the area under the curve (AUC) of serum viral loads over the post-infection period using the trapezoidal rule (38). Ct values were normalized by the cut-off value (40 Ct), and individual AUC values were calculated based on normalized Ct data. Group comparisons were performed using Student’s *T* test. The relative percentage of viremia reduction was calculated as follows: RPVR = [1-(average AUC of VC/average AUC NVC group)] x 100.

Clinical signs and viral loads in serum between experimental groups were compared using the Chi-square test or Fisher’s exact test, depending on data characteristics. The normality of viremia and ELISA data was assessed using the Shapiro–Wilk test before ANOVA. As the data were normally distributed, group differences were analyzed using ANOVA. The mean and standard deviation of viremia and ELISA results were analyzed using analysis of variance (ANOVA) to assess statistical differences among groups. Differences between groups were considered statistically significant at *p* < 0.05. Results are presented as mean ± standard deviation (SD) or as median with range, depending on the data distribution.

## 3 Results

### 3.1 Clinical signs

**Figure 3:**
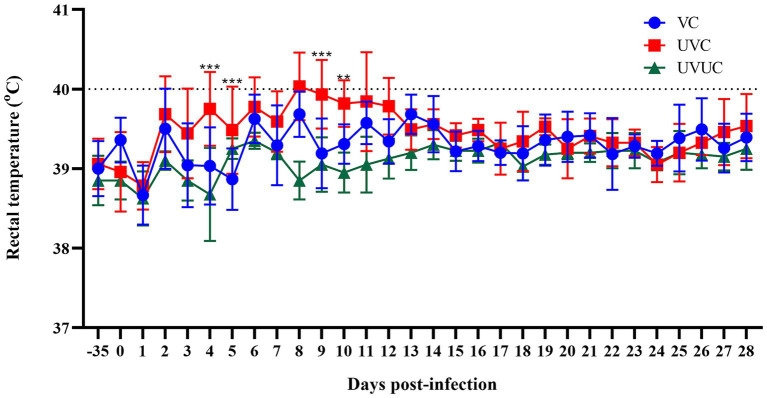
Body temperature was recorded daily. *, **, and *** indicate statistically significant differences at *p* < 0.05, *p* < 0.01, and *p* < 0.001, respectively. VC group, vaccinated and challenged; UVC group, unvaccinated and challenged; UVUC group, unvaccinated and unchallenged.

No clinical abnormalities were detected in any of the animals prior to the viral challenge, indicating that all pigs were clinically healthy. Following challenge with the virulent HP-PRRSV strain, all infected groups developed clinical signs, though the severity varied among groups. Rectal temperature monitoring revealed difference among experimental groups. UVC pigs developed marked pyrexia (>40.0 °C) starting at 2 dpi, with peak temperatures recorded at 8 dpi. In contrast, pigs in the VC group maintained the body temperature below 40.0 °C throughout the observation period. Statistical analysis demonstrated significant differences in mean rectal temperatures between VC and UVC groups at acute period of diseases as 4, 5, 9 and 10 dpi (*p* < 0.01; Figure 3).

**Figure 4:**
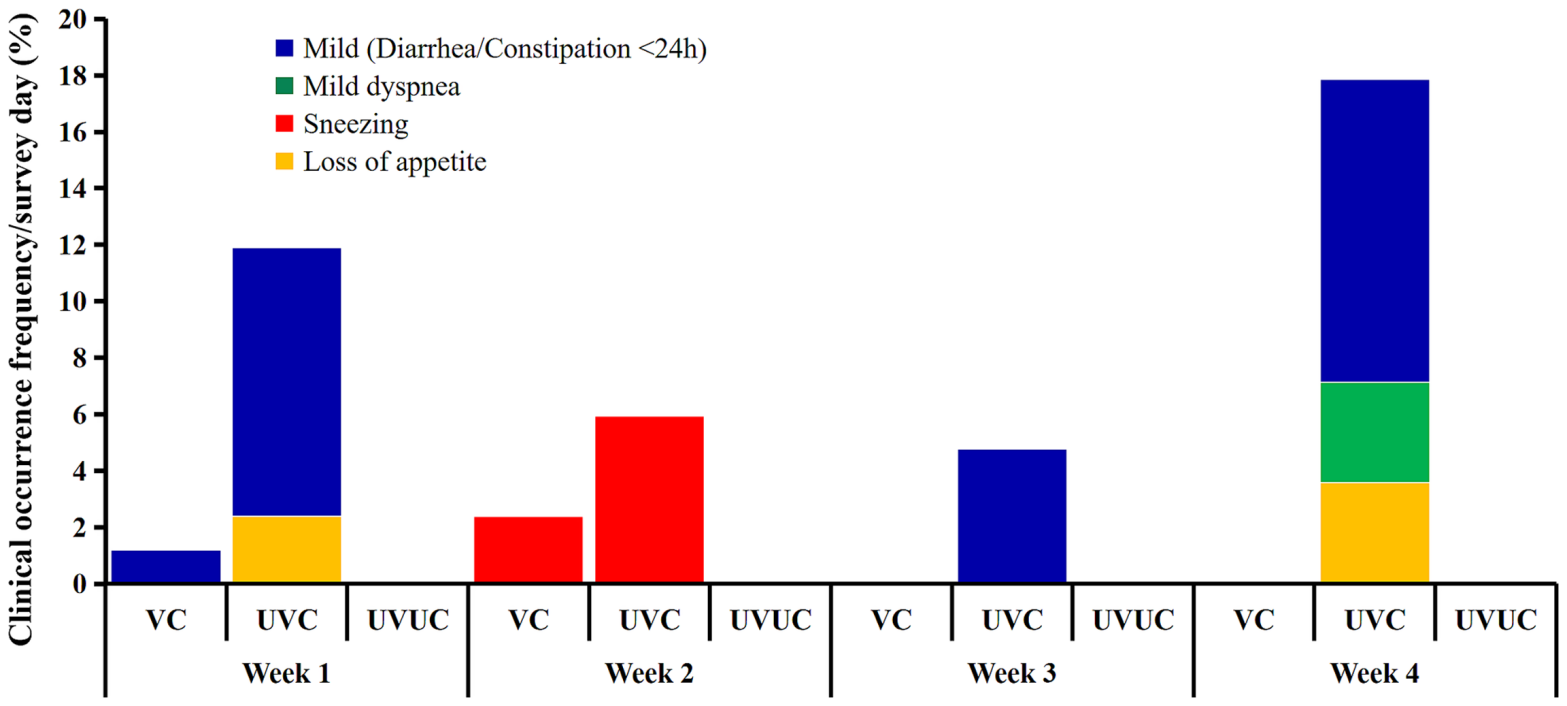
Frequency of clinical signs in weaned pigs following the 4-week post PRRSV challenge. VC group, vaccinated and challenged; UVC group, unvaccinated and challenged; UVUC group, unvaccinated and unchallenged.

Among the clinical signs observed after challenge, mild diarrhea was the most frequently recorded sign across UVC groups. In addition, several pigs exhibited mild labored breathing, occasional sneezing, and transient inappetence. In VC pigs, clinical signs appeared later (approximately 4 dpi) and resolved rapidly within several days. In contrast, UVC pigs developed earlier clinical signs (around 3 dpi), and the symptoms persisted until the end of the experiment. No neurological or dermatological abnormalities were observed in any animal during the study period. The incidence and duration of clinical manifestations were markedly reduced in VC pigs compared with UVC controls. Most animals in the VC group remained clinically normal or exhibited only transient, mild respiratory signs that subsided within 1–2 days without progression. Conversely, UVC pigs developed more persistent and pronounced symptoms, including prolonged diarrhea and moderate respiratory distress lasting up to 7–10 days. Quantitative analysis of daily clinical records confirmed a significantly lower frequency and severity of clinical signs in VC group compared with UVC group (Figure 4).

### 3.2 Lung lesions

**Figure 5:**
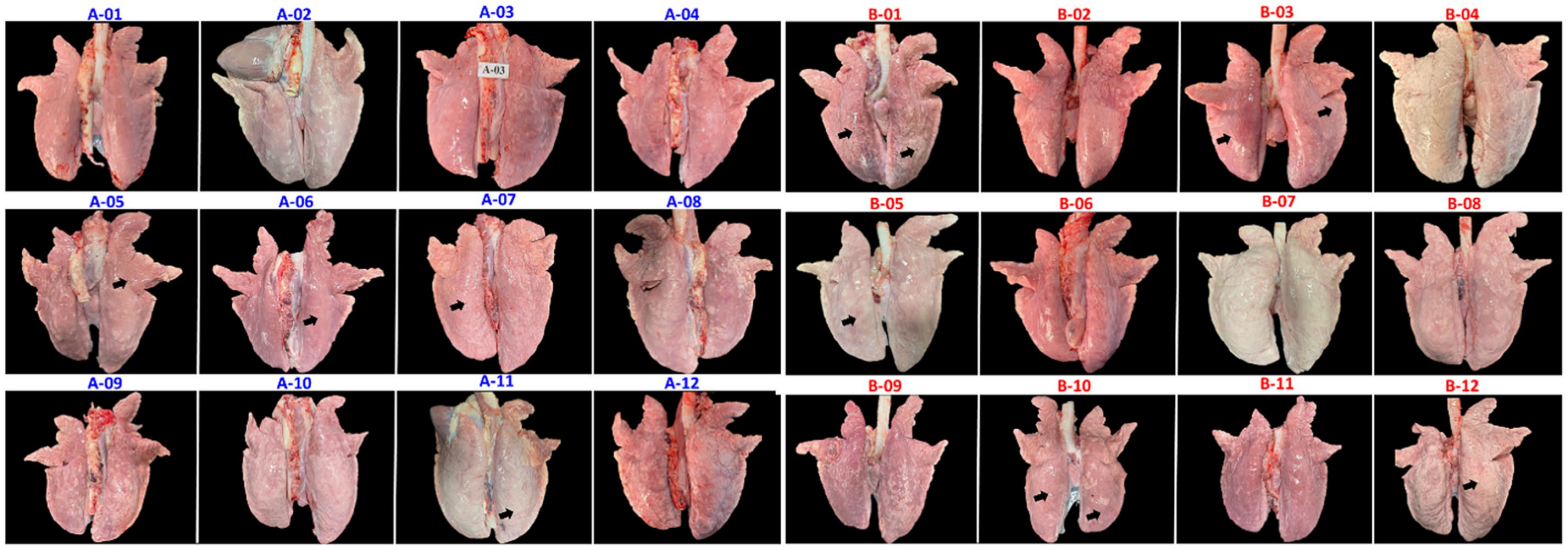
Gross lung lesions in pigs from the two experimental groups are shown: VC group **(A01–A12)** and UVC group **(B01–B12)**. Arrows highlight areas of interstitial pneumonia and petechial hemorrhages within the lung tissue.

**Figure 6:**
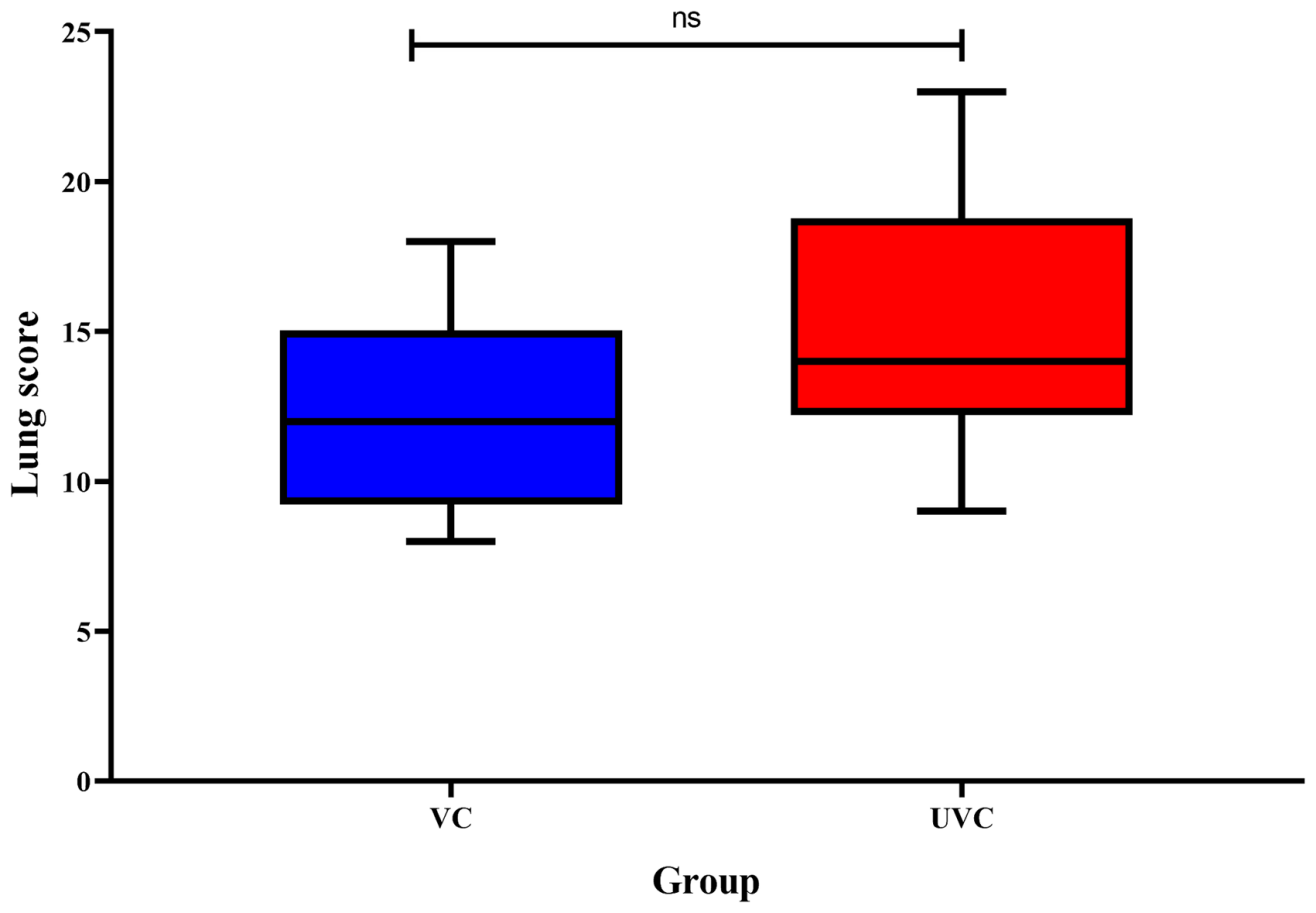
Macroscopic lung lesion scores at 28 days post-infection. “ns” indicates not significant (*p* > 0.05). VC group, vaccinated and challenged; UVC group, unvaccinated and challenged.

Lung lesions were assessed at the end of the experiment. Necropsy findings revealed macroscopic pneumonic lesions in both challenged groups, which appeared more extensive in the UVC group (Figure 5). The predominant pathological changes included interstitial pneumonia and petechial hemorrhages. The mean lung lesion score in the VC group (12.66 ± 3.39) was lower than that of the UVC group (15.5 ± 4.49); however, this difference was not statistically significant (*p* > 0.05) (Figure 6).

### 3.3 Histopathological findings in lung sections

**Figure 7:**
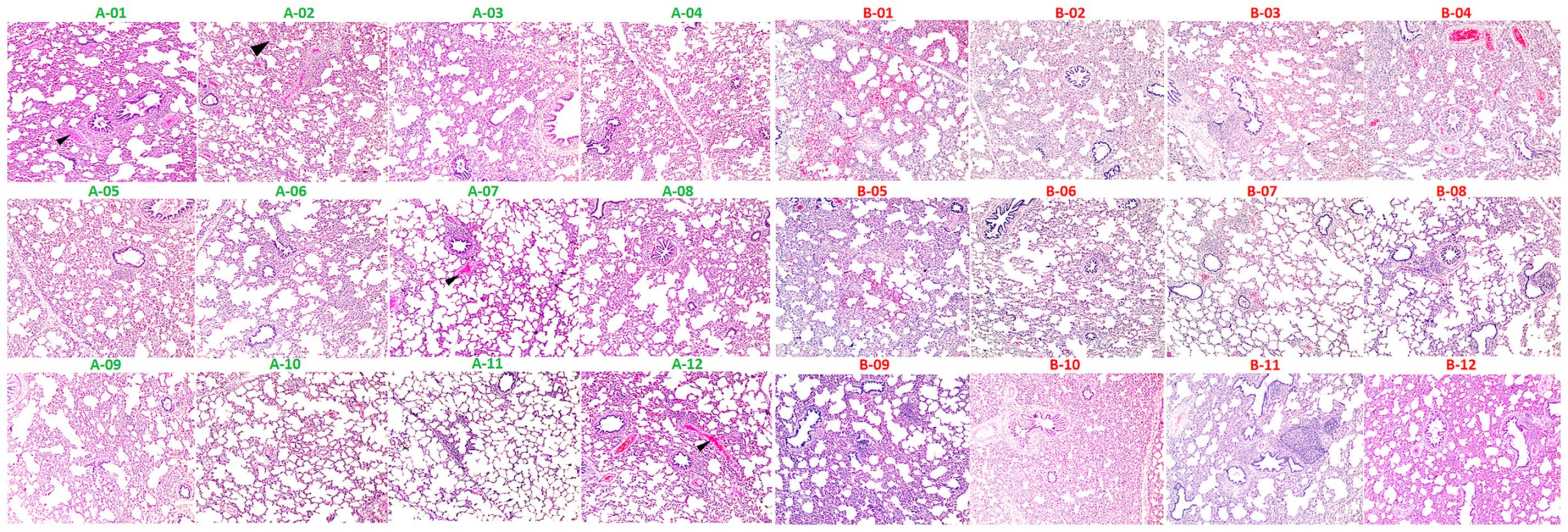
Microscopic lung lesions in pigs from two experimental groups are shown: the VC group **(A01–A12)** and the UVC group **(B01–B12)**. Arrows highlight areas of interstitial pneumonia and petechial hemorrhages in the lung tissue.

In the VC group (sections A-01 to A-12), the pulmonary parenchyma exhibited largely preserved alveolar architecture. Alveolar lumina remained patent, and only mild thickening of the alveolar septa was observed. Scattered perivascular and peribronchiolar mononuclear cell infiltrates were present, but the overall inflammatory cell density was low. Occasional foci demonstrated mild capillary congestion within alveolar walls; however, there was no evidence of diffuse hemorrhage, epithelial necrosis, or fibrin deposition. The pulmonary lesions were classified as mild to moderate interstitial pneumonia, characterized by minimal structural disruption and well-aerated parenchyma (Figure 7).

**Figure 8:**
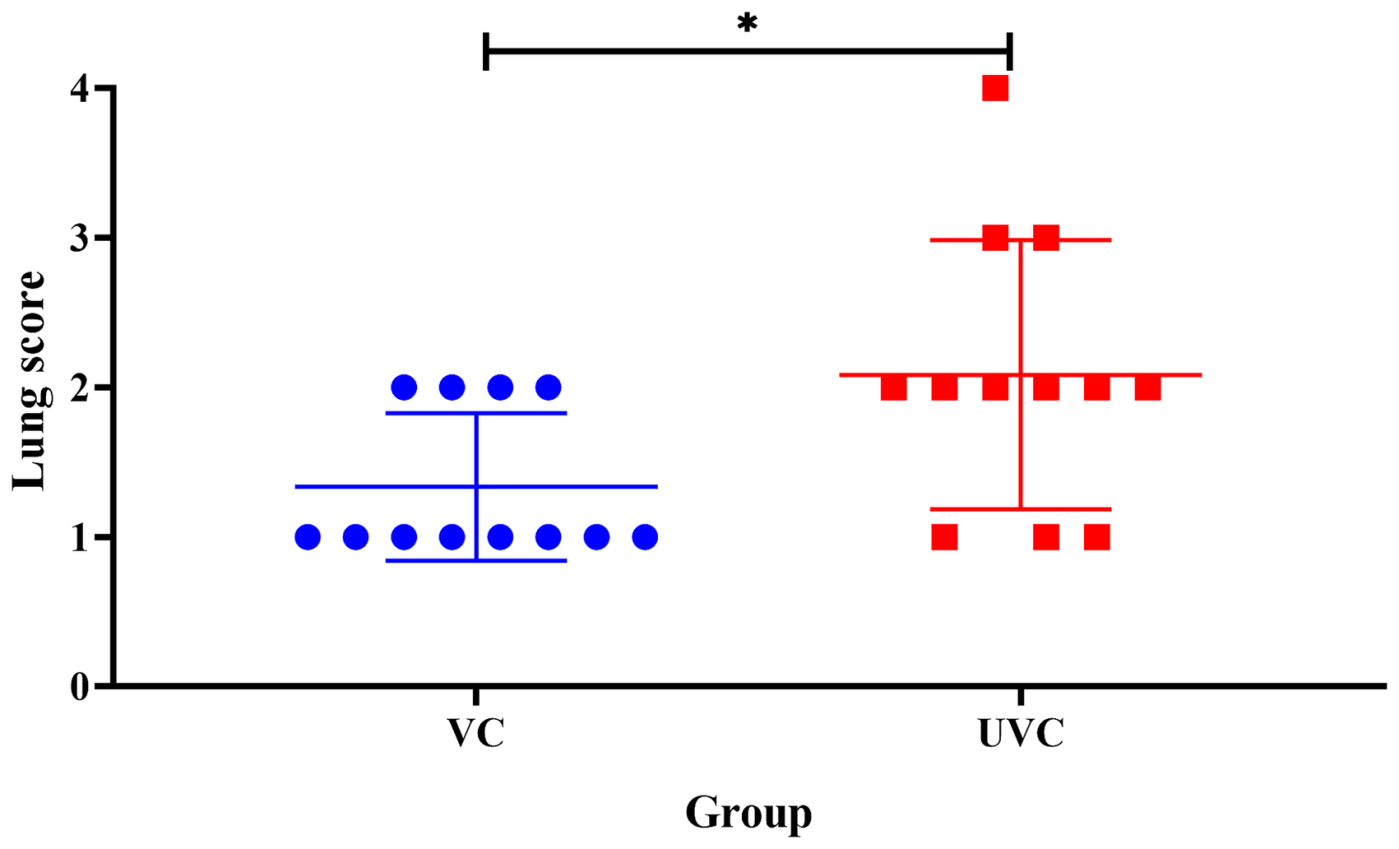
Microscopic lung lesion scores in pigs from the vaccinated and challenged (VC) group and the unvaccinated and challenged (UVC) group. Each dot represents an individual animal, and horizontal bars indicate the median values. Lesion scores were defined as follows: 0, no microscopic lesions; 1, mild interstitial pneumonia; 2, moderate multifocal interstitial pneumonia; 3, moderate diffuse interstitial pneumonia; and 4, severe interstitial pneumonia.

In contrast, the UVC group (sections B-01 to B-12) displayed pronounced histopathological alterations. The alveolar septa were markedly thickened, accompanied by multifocal atelectasis and areas of consolidation in which alveolar lumina were narrowed or obliterated. The interstitial and perivascular regions were densely infiltrated with mononuclear inflammatory cells, occasionally forming nodular aggregates. Widespread vascular congestion and hemorrhage were evident, with erythrocyte extravasation into alveolar and interstitial spaces. Mild alveolar edema, bronchiolar epithelial hyperplasia, and prominent peribronchial inflammation were also observed (Figure 8). The VC group showed a significantly lower median lung lesion score (1.00) compared with the UVC group (2.00) (*p* < 0.05).

### 3.4 Histopathological findings in pulmonary lymph nodes

**Figure 9:**
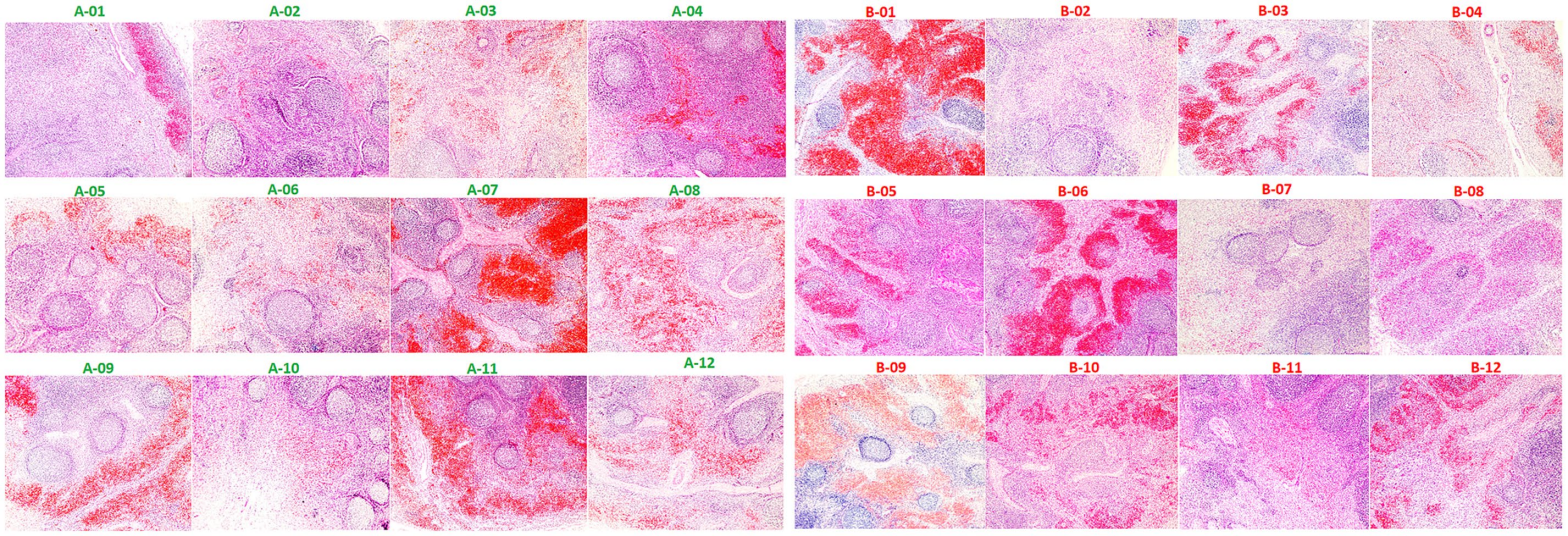
Microscopic pulmonary lymph node lesions in pigs from two experimental groups are shown: the VC group **(A01–A12)** and the UVC group **(B01–B12)**.

In the VC group, most pigs (A-02, A-04, A-05, A-06, A-09, A-10, A-12) exhibited relatively well-preserved lymph node architecture. Lymphoid follicles were clearly delineated, displaying mildly hyperplastic germinal centers, and vascular congestion was focal and of mild to moderate intensity. In a subset of pigs (A-01, A-03, A-07, A-08, A-11), lesions were more pronounced, characterized by patchy to regionally diffuse hemorrhage and partial disruption of the nodal framework. Notably, sections A-07 and A-11 showed extensive hemorrhagic areas involving a large portion of the parenchyma, whereas A-01, A-03, and A-08 exhibited focal necrotic or degenerative changes and dilated sinuses containing erythrocytes. Importantly, none of the samples showed complete architectural effacement or diffuse necrosis. Overall, lesions ranged from mild to locally severe, predominantly representing reactive inflammatory changes associated with vascular congestion (Figure 9).

In the UVC group (sections B-01 to B-12), histopathological lesions were generally more extensive and severe. Samples B-01, B-03, B-05, B-06, B-09, and B-12 exhibited diffuse hemorrhage, marked effacement of nodal architecture, and sinuses densely packed with erythrocytes, accompanied by stromal edema and intense inflammatory cell infiltration. Sections B-04, B-06, B-08, B-10, and B-11 showed moderate to severe hemorrhage with indistinct cortico-medullary demarcation and multifocal areas of lymphoid degeneration or depletion. By contrast, samples B-02 and B-07 were comparatively less affected, retaining partial follicular organization with focal vascular congestion and a mild increase in mononuclear cells. Overall, moderate to severe lesions predominated in the UVC group, characterized by extensive hemorrhage, stromal edema, lymphoid depletion, and near-complete architectural effacement in multiple sections.

### 3.5 Viral load in blood

Whole-blood RT-qPCR analysis revealed that no viremia was detected in any pig from either group prior to vaccination (−35 dpi) (Table 1). Fourteen days after vaccination (−21 dpi), viremia corresponding to the PRRSV vaccine strain was detected in 11 out of 12 pigs (91.7%) in the VC group, with relatively high Ct values (28.34 ± 4.28). At the day of challenge (0 dpi), 11 of 12 pigs in the VC group remained positive for the PRRSV vaccine strain, whereas all 12 pigs in the UVC group remained negative for PRRSV vaccine strain from −35 dpi to 0 dpi.

**Table 1:** Viremia in experimental pigs in various groups.

DPI	EU						NA					
	VC		UVC		UVUC		VC		UVC		UVUC	
	n/N	Mean ± SD	n/N	Mean ± SD	n/N	Mean ± SD	n/N	Mean ± SD	n/N	Mean ± SD	n/N	Mean ± SD
−35 dpi	0/12	/	0/12	/	0/4	/	0/12	/	0/12	/	0/4	/
−21 dpi	11/12	28.34 ± 4.28	0/12	/	0/4	/	1/12	35.36 ± 0	0/12	/	0/4	/
0 dpi	11/12	33.96 ± 3.25	0/12	/	0/4	/	0/12	/	0/12	/	0/4	/
7 dpi	5/12	33.61 ± 2.64	0/12	/	0/4	/	10/12	30.09 ± 3.69^a^	12/12	25.45 ± 1.39^b^	0/4	/
14 dpi	7/12	34.42 ± 3.76	0/12	/	0/4	/	10/12	29.58 ± 4.57^a^	12/12	25.39 ± 2.54^a^	0/4	/
21 dpi	6/12	36.36 ± 1.73	0/12	/	0/4	/	8/12	33.46 ± 3.16^a^	10/12	31.63 ± 3.42^a^	0/4	/
28 dpi	1/12	31.768 ± 0	0/12	/	0/4	/	4/12	33.034 ± 3.6^a^	6/12	34.86 ± 2.11^a^	0/4	/

*a, b are letters that indicate statistically significant differences at *p* < 0.05. VC group, vaccinated and challenged; UVC group, unvaccinated and challenged. The table shows the mean Ct values and the proportion of RT-qPCR PRRSV-positive animals at each time point.

**Figure 10:**
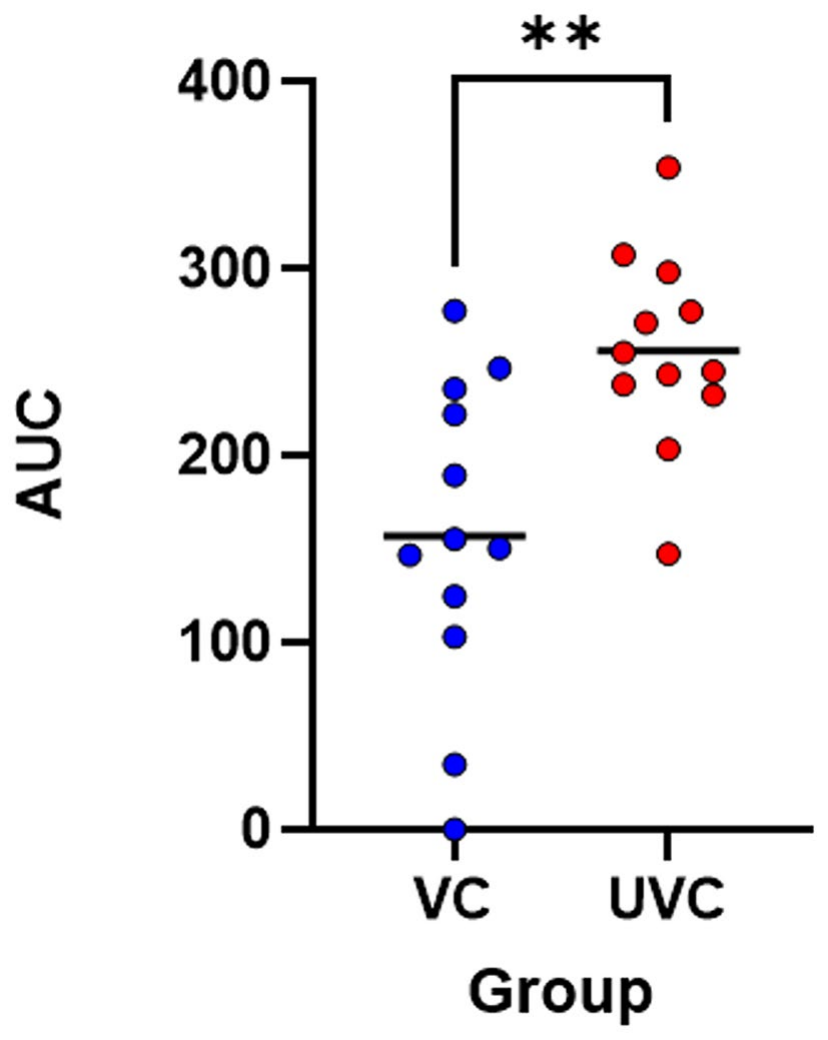
Dot plot of the individual AUC values of PRRSV viremia during the post-infection period. The horizontal bars represent the average. Asterisks indicate a statically significant difference (Student’s *T* test; *p* < 0.01).

In the VC group, approximately half of the pigs exhibited persistent EU strain viremia up to 21 days after vaccination, indicating continued replication of the vaccine virus. In contrast, pigs in the UVC group showed detectable viremia for NA strains after challenge, which persisted up to 28 dpi. Both VC and UVC groups developed PRRSV-2 viremia after challenge; however, the proportion of positives and viral loads (higher Ct) were lower in VC at 7–14 dpi, with persistence reduced by 28 dpi. The AUC analysis revealed that the cumulative viremia in the VC group was significantly lower compared to the UVC group resulting in a RPVR of 38.58% (*p* < 0.01; Figure 10).

### 3.6 Serology

**Figure 11:**
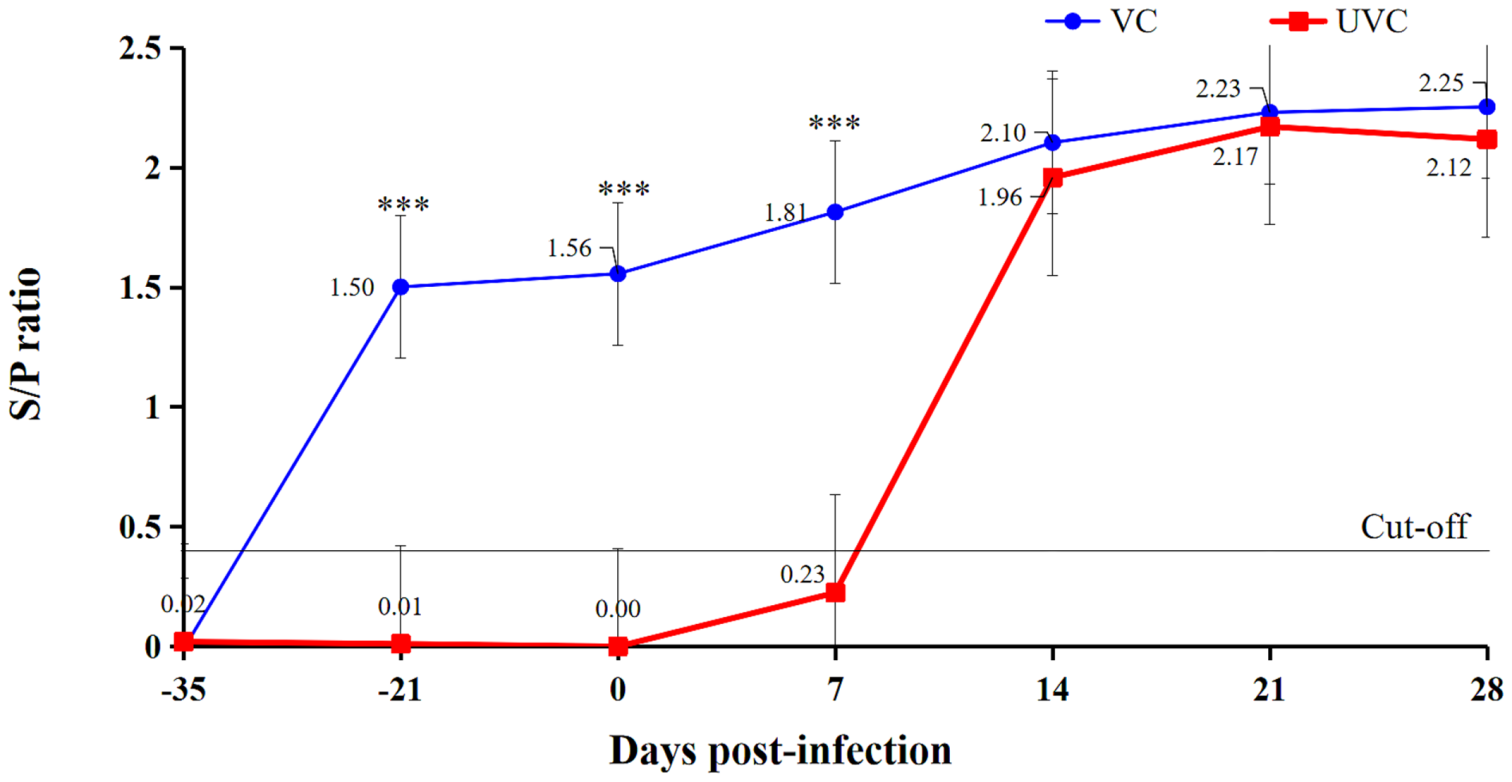
Seroconversion as determined by ELISA. The graph shows the average S/P ratio values for each group as determined using the Idexx PRRSV ELISA during the vaccination and challenge phases. *, **, ***: Statistically significant differences at *p* < 0.05; *p* < 0.01; and *p* < 0.001, respectively. UVUC group, unvaccinated and unchallenged; VC group, vaccinated and challenged; UVC group, unvaccinated and challenged.

The experimental pigs were all PRRSV seronegative at the beginning of the study. Vaccination induced seroconversion in most animals between 14 and 21-days post administration of the vaccine. In the vaccinated pigs, inoculation with the HP-PRRSV-2 (L8E) virus resulted in a significant increase in the S/P ratios by 14 dpi (1.56 ± 0.63 before inoculation vs. 2.1 ± 0.31 at 14 dpi, *p* < 0.05; Figure 11). In the UVC group, inoculation with the HP-PRRSV-2 induced rapid seroconversion by 14 dpi in all pigs. No seroconversion was detected in the UVUC group, as all pigs remained sero-negativity at both the initial and throughout 28 dpi.

### 3.7 Mortality (%)

All pigs survived until the end of the study, and no mortality was observed during the experiment.

### 3.8 Growth of performance

**Figure 12:**
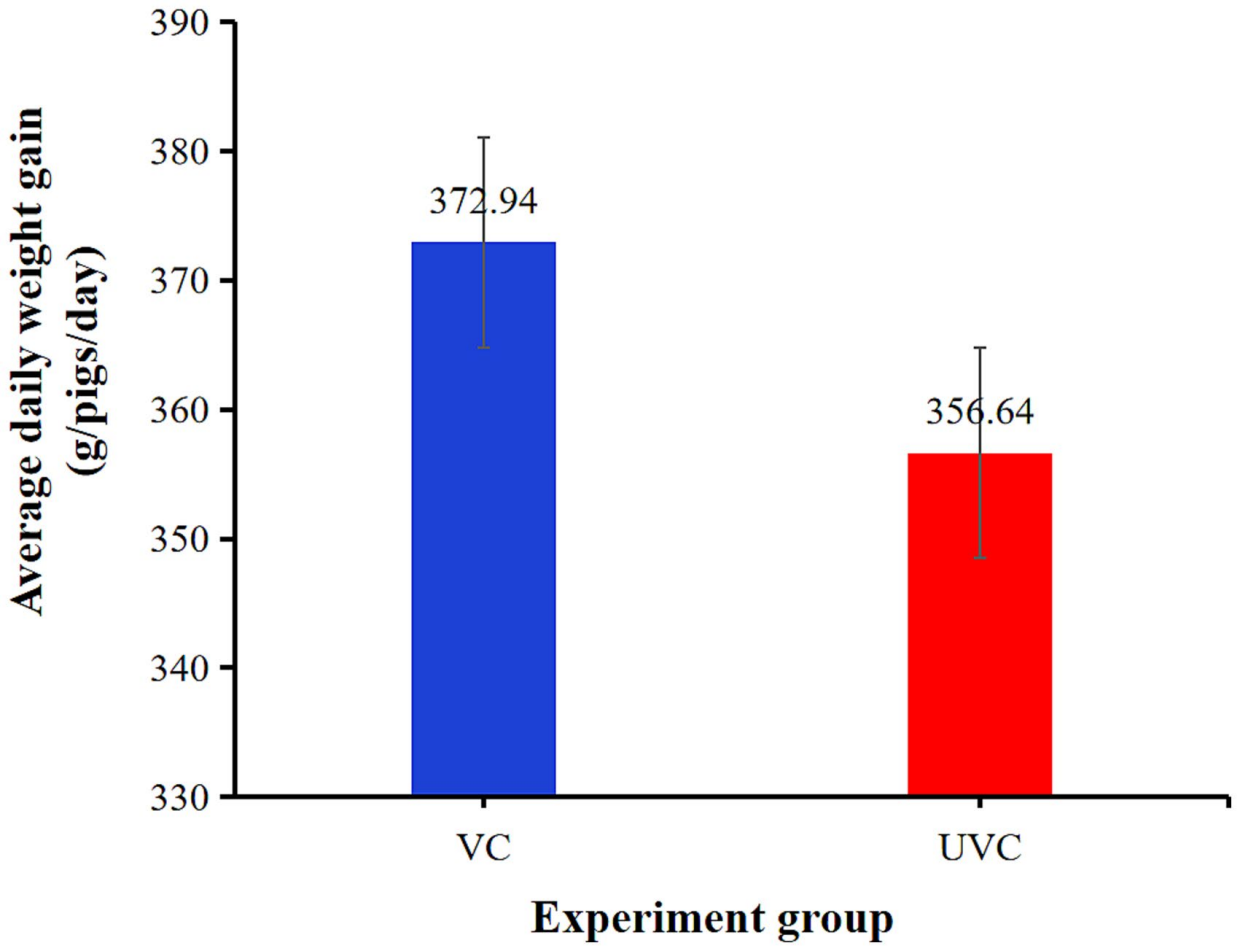
Growth performance of piglets in the study: Average daily gain of piglets throughout the entire experimental period. VC group, vaccinated and challenged; UVC group, unvaccinated and challenged.

Over the trial period, pigs in the VC group increased from 5.29 to 28.78 kg, corresponding to a total BW gain of 23.49 kg. In the UVC group, BW increased from 5.64 to 28.11 kg, corresponding to a total BW gain of 22.47 kg. The ADG of pigs in the VC group (372.94 g) was numerically higher than that of the UVC group (356.64 g), but the difference was not statistically significant (*p* > 0.05; Figure 12).

## 4 Discussion

Effective control of PRRS remains a major challenge in modern swine production and requires a multidimensional approach in which immune protection plays a pivotal role. Vaccination continues to be the most reliable and practical strategy for establishing herd immunity. However, in several regions where both PRRSV-1 and PRRSV-2 co-circulated, effective control is particularly challenging, as a single vaccine strain rarely induces broad heterologous protection (39).

Previous research has indicated that PRRSV-1 MLV vaccines may induce partial cross-protection against heterologous PRRSV-2 strains. For example, Roca et al. (40), demonstrated that intramuscular (IM) vaccination with a PRRSV-1 MLV significantly reduced mortality, pyrexia duration, lung lesion severity, and viremia following HP-PRRSV-2 challenge. Similarly, Madapong et al. (32), reported reduced viremia and pulmonary lesions following dual PRRSV-1 and PRRSV-2 challenge in MLV-vaccinated pigs. However, cross-protection conferred by PRRS vaccines remains limited and inconsistent, even between homologous species, and is largely strain-dependent. Recent evidence has demonstrated reduced efficacy of a VR-2332-based modified live vaccine against heterologous PRRSV-2 NADC30-like strain HNjz15 (41). Accordingly, the use of a HP-PRRSV-2 strain in the present study represents a stringent experimental model to evaluate vaccine efficacy against virulent PRRSV strains.

The results of this study demonstrated that the PRRSV-1 MLV vaccine administered via the ID route provided measurable, partial protection against HP-PRRSV-2 challenge. Vaccinated pigs displayed significantly lower clinical scores and body temperatures compared with unvaccinated controls. Notably, vaccinated pigs did not display any prolonged or recurrent febrile responses, indicating that vaccination effectively prevented the hyperthermia typically associated with acute HP-PRRSV-2 infection. Clinical signs in the VC group were generally mild and transient, whereas the UVC group exhibited pronounced respiratory and gastrointestinal signs. These results indicate that vaccination mitigated disease severity, consistent with previous reports that PRRSV-1 MLV vaccination can attenuate clinical manifestations following heterologous PRRSV-2 infection (40). Regarding viremia, all pigs developed detectable viral RNA after challenge; however, the magnitude and duration of viremia were markedly reduced in vaccinated animals. At 7 dpi, 100% of control pigs were strongly positive for PRRSV, whereas only 83.3% of vaccinated pigs tested positive. By 28 dpi, viremia persisted in 50% of control pigs but only 33.3% of vaccinated pigs. Moreover, vaccinated pigs showed higher Ct values, indicating lower viral loads. These results suggest that vaccination delayed viral replication and reduced systemic viral burden but did not fully prevent infection. These observations align with the known limitations of current PRRSV vaccines, which confer strong homologous protection but only partial or inconsistent heterologous protection (26, 31). Previous studies have demonstrated that the level of heterologous PRRSV cross-protection is frequently associated with the genetic relationship between vaccine and challenge strains, particularly within ORF5 encoding the major envelope glycoprotein GP5, which contains important neutralizing epitopes (42, 43). Amino acid variability within GP5 may contribute to incomplete cross-protection because mutations in immunologically relevant epitopes can alter antibody recognition and virus neutralization (43, 44). Consequently, current PRRSV modified live vaccines generally reduce clinical severity and viral load but often provide incomplete heterologous protection due to the extensive genetic and antigenic diversity among circulating PRRSV strains (27, 45). The importance of GP5 sequence similarity in heterologous protection has been further supported by studies reporting broader protection against PRRSV strains sharing approximately 85–93% GP5 amino acid identity (46).

Although the challenge isolate was classified as a highly pathogenic PRRSV-2 strain, no mortality was observed in either experimental group. This outcome may be associated with several experimental factors, including the controlled housing environment, absence of secondary infections, intensive animal care, relatively short observation period, and the high-health status of the pigs used in this study. Previous studies have demonstrated that the severity and mortality of HP-PRRSV infection can vary considerably depending on host condition, challenge protocol, co-infections, environmental stressors, and management conditions (3, 47, 48). In field outbreaks, mortality associated with HP-PRRSV is frequently exacerbated by concurrent infections and environmental factors that are difficult to fully reproduce under controlled experimental settings (3, 27).

Vaccinated pigs seroconverted earlier and maintained higher titer throughout the experiment. Following challenge, antibody levels increased in both groups, but the VC group exhibited significantly higher titers at 7 dpi. This indicates that ID vaccination with the PRRSV-1 MLV vaccine successfully established a primed immune status, enabling a faster and stronger anamnestic response upon exposure to HP-PRRSV-2. These results reinforce the ability of MLV vaccines to stimulate cross-reactive humoral immunity that mitigates disease progression despite genetic divergence between vaccine and field strains (49–52). These findings are consistent with previous heterologous PRRSV challenge studies reporting that modified live vaccines may reduce viremia and pulmonary lesion severity despite incomplete prevention of infection when genetically divergent PRRSV strains are involved (30, 32, 53).

Histopathological examination revealed that vaccinated pigs exhibited a significant reduction of 36% in lung lesion scores compared with the UVC group. Lesions in vaccinated pigs were predominantly mild to moderate interstitial pneumonia characterized by limited alveolar septal thickening and scattered inflammatory infiltration. In contrast, unvaccinated pigs showed multifocal to diffuse interstitial pneumonia with alveolar collapse, thickened interstitium, and perivascular cuffing. These findings demonstrate that vaccination effectively reduced the severity of pulmonary inflammation and tissue damage, consistent with prior reports that PRRSV-1 MLV can alleviate bronchointerstitial pneumonia caused by heterologous HP-PRRSV-2 infection (40, 32). The milder lung pathology observed here supports the hypothesis that partial cross-protection induced by heterologous MLV vaccines is primarily mediated through cellular rather than sterilizing immunity (54).

With regard to growth performance, vaccinated pigs demonstrated numerically higher ADG and maintained feed intake throughout the post-infection stage, whereas unvaccinated pigs showed reduced weight gain during the febrile phase. Although differences in growth performance did not reach statistical significance, the observed numerical trends were consistent with the reduced clinical severity and faster recovery observed in vaccinated animals, as systemic inflammation and prolonged fever are known to impair feed efficiency and nutrient utilization (28). This may be associated with the relatively short experimental duration, limited sample size, and the controlled experimental setting, which may reduce the sensitivity for detecting production-related differences. Previous PRRSV vaccine studies have similarly reported that virological and pathological reductions are more consistently observed than significant improvements in growth performance during short-term experimental challenges (53, 55) These results indicate a tendency toward improved growth performance in vaccinated PRRSV-1 pigs during the post-infection period following heterologous PRRSV-2 challenge.

Despite providing valuable insights into the heterologous protection induced by the PRRSV-1 MLV against HP-PRRSV-2 strain, this study has several limitations that should be acknowledged. The relatively small sample size may have restricted the statistical power to detect subtle differences among groups. Only one HP-PRRSV-2 isolate (lineage L8E) was used for the challenge, which may not fully capture the genetic diversity of circulating field strains. Viral quantification was confined to serum samples, without assessing tissue viral loads, and the observation period was limited to 28 days post-infection. These factors may have constrained the comprehensive evaluation of viral kinetics and long-term immune responses.

Taken together, these findings reinforce that while PRRSV-1 MLV vaccination does not fully prevent replication of heterologous HP-PRRSV-2 strains, it markedly mitigates disease severity at clinical, virological, and pathological levels. Additionally, the intradermal route of administration has been reported to offer practical and immunological advantages over traditional intramuscular delivery, including reduced animal stress, lower risk of injection-site contamination, and enhanced biosafety (56, 54).

## 5 Conclusion

Intradermal vaccination with a commercial PRRSV-1 MLV reduced clinical signs, viremia, and enhanced humoral responses against heterologous HP-PRRSV-2 challenge. These findings indicate that intradermal vaccination of a PRRSV-1 MLV cross-protects against the highly pathogenic PRRSV-2 infection and represent may serve as a useful tool component for PRRS control programs.

## Data Availability

The datasets presented in this study can be found in online repositories. The names of the repository/repositories and accession number(s) can be found in the article/supplementary material.
